# Supplementary material for: The polarizing impact of numeracy, economic literacy, and science literacy on the perception of immigration
Source: PLoS One. 2022 Oct 7;17(10):e0274680. doi: 10.1371/journal.pone.0274680 (PMC9543957; doi:10.1371/journal.pone.0274680)
Supplement: S2 Table — Selected sample (absolute frequencies and percentages) stratified by gender (female/male), age groups (18–35, 36–55, and 56–80 years), and macro-area of residence (South-West, Center-North, North-East). (DOCX) [file pone.0274680.s002.docx]

**Table S2. Selected sample**. Selected sample (absolute frequencies and percentages) stratified by gender (female/male), age groups (18-35, 36-55, and 56-80 years), and macro-area of residence (South-West, Center-North, North-East).

|  |  | **Absolute frequency** | **%** |
| --- | --- | --- | --- |
|  | Female | 1,031 | 51.3 |
| **Gender** | Male | 977 | 48.7 |
|  | Total | 2,008 | 100 |
|  |  |  |  |
|  | 18 – 35 | 530 | 26.4 |
| **Age** | 36 – 55 | 747 | 37.2 |
|  | 56 – 80 | 731 | 36.4 |
|  | Total | 2,008 | 100 |
|  |  |  |  |
|  | South – West | 625 | 31.1 |
| **Area** | Center – North | 900 | 44.8 |
|  | North – East | 483 | 24.1 |
|  | Total | 2,008 | 100 |
