# Supplementary material for: The polarizing impact of numeracy, economic literacy, and science literacy on the perception of immigration
Source: PLoS One. 2022 Oct 7;17(10):e0274680. doi: 10.1371/journal.pone.0274680 (PMC9543957; doi:10.1371/journal.pone.0274680)
Supplement: S3 Table — Response rate (absolute frequencies and percentages) stratified by gender (female/male), age groups (18–35, 36–55 and 56–80 years), and macro-area of residence (South-West, Center-North, North-East). (DOCX) [file pone.0274680.s003.docx]

**Table S3. Response rate**. Response rate (absolute frequencies and percentages) stratified by gender (female/male), age groups (18-35, 36-55 and 56-80 years), and macro-area of residence (South-West, Center-North, North-East).

|  |  | **Absolute frequency** | **%** |
| --- | --- | --- | --- |
|  | Female | 270 | 26.2 |
| **Gender** | Male | 281 | 28.8 |
|  | Total | 551 | 100 |
|  |  |  |  |
|  | 18 – 35 | 108 | 20.4 |
| **Age** | 36 – 55 | 188 | 25.2 |
|  | 56 – 80 | 255 | 34.9 |
|  | Total | 551 | 100 |
|  |  |  |  |
|  | South – West | 178 | 28.5 |
| **Area** | Center – North | 234 | 26 |
|  | North – East | 139 | 28.8 |
|  | Total | 551 | 100 |
