# Supplementary material for: The polarizing impact of numeracy, economic literacy, and science literacy on the perception of immigration
Source: PLoS One. 2022 Oct 7;17(10):e0274680. doi: 10.1371/journal.pone.0274680 (PMC9543957; doi:10.1371/journal.pone.0274680)
Supplement: S5 Table — Descriptive statistics for cultural worldviews. (DOCX) [file pone.0274680.s005.docx]

**Table S5. Worldviews Descriptives**. Descriptive statistics for cultural worldviews

|  | Sample mean | Standard deviation | Median | Minimun | Maximum | Number of observations | Missing |
| --- | --- | --- | --- | --- | --- | --- | --- |
| IINTRSTS | 3.17 | 0.91 | 3 | 1 | 5 | 524 | 27 |
| CHARM | 2.49 | 0.98 | 2 | 1 | 5 | 518 | 33 |
| IPROTECT | 2.67 | 1.08 | 2 | 1 | 5 | 524 | 27 |
| IPRIVACY | 3.38 | 0.98 | 3 | 1 | 5 | 523 | 28 |
| CPROTECT | 3.13 | 1.10 | 3 | 1 | 5 | 526 | 25 |
| CLIMCHOI | 3.24 | 1.07 | 3 | 1 | 5 | 529 | 22 |
| HEQUAL | 2.41 | 1.17 | 2 | 1 | 5 | 533 | 18 |
| EWEALTH | 1.80 | 0.84 | 2 | 1 | 5 | 543 | 8 |
| ERADEQ | 1.88 | 0.94 | 2 | 1 | 5 | 539 | 12 |
| EDISCRIM | 2.12 | 0.97 | 2 | 1 | 5 | 541 | 10 |
| HREVDIS2 | 2.44 | 1.18 | 2 | 1 | 5 | 535 | 16 |
| HFEMININ | 2.99 | 1.25 | 3 | 1 | 5 | 537 | 14 |
| Worldview orientation | 2.63 | 0.45 | 2.54 | 1.17 | 4.25 | 548 | 3 |
