# Supplementary material for: The polarizing impact of numeracy, economic literacy, and science literacy on the perception of immigration
Source: PLoS One. 2022 Oct 7;17(10):e0274680. doi: 10.1371/journal.pone.0274680 (PMC9543957; doi:10.1371/journal.pone.0274680)
Supplement: S7 Table — Descriptive statistics for perception of immigration. (DOCX) [file pone.0274680.s007.docx]

**Table S7. Perception of immigration Descriptives.** Descriptive statistics for perception of immigration

|  | Sample mean | Standard deviation | Median | Minimun | Maximum | Number of observations | Missing |
| --- | --- | --- | --- | --- | --- | --- | --- |
| Q1 | 3.31 | 1.11 | 4 | 1 | 5 | 545 | 6 |
| Q2 | 3.57 | 1.17 | 4 | 1 | 5 | 542 | 9 |
| Q3 | 2.73 | 1.23 | 2 | 1 | 5 | 538 | 13 |
| Q4 | 3.44 | 1.10 | 4 | 1 | 5 | 537 | 14 |
| Q5 | 2.27 | 1.04 | 2 | 1 | 5 | 547 | 4 |
| Q6 | 3.55 | 1.25 | 4 | 1 | 5 | 527 | 24 |
| Q7 | 3.53 | 1.13 | 4 | 1 | 5 | 529 | 22 |
| Q8 | 3.07 | 1.32 | 3 | 1 | 5 | 505 | 46 |
| Q9 | 3.65 | 1.10 | 4 | 1 | 5 | 529 | 22 |
| Q10 | 2.50 | 1.26 | 3 | 1 | 5 | 451 | 100 |
| Q11 | 3.47 | 1.00 | 4 | 1 | 5 | 534 | 17 |
| Q12 | 2.97 | 1.23 | 3 | 1 | 5 | 498 | 53 |
| Q13 | 2.83 | 0.96 | 3 | 1 | 5 | 530 | 21 |
| *y*^1^ | 3.16 | 0.75 | 3.23 | 1.15 | 4.69 | 549 | 2 |

^1^The response variable (perception of immigration) of our models.
