# Supplementary material for: The polarizing impact of numeracy, economic literacy, and science literacy on the perception of immigration
Source: PLoS One. 2022 Oct 7;17(10):e0274680. doi: 10.1371/journal.pone.0274680 (PMC9543957; doi:10.1371/journal.pone.0274680)
Supplement: S8 Table — Items used in the survey to measure numeracy [84]. (DOCX) [file pone.0274680.s008.docx]

**Table S8. Numeracy items.** Items used in the survey to measure numeracy (84).

| Numeracy |
| --- |
| Q16 (CRT). If it takes five machines 5 minutes to make five widgets, how long would it take 100 machines to make 100 widgets? (100, 20, 10, or **5 minutes**)  Q15 (CRT). A notebook and a pen cost € 1.80 in total. The notebook costs € 1.00 more than the pen. How much does the pen cost? (€0.80, **€0.40**, €0.20, or € 1.00)^a^  Q3. In a lottery, the chance of winning a car is 1 in 1000. What percentage of tickets in that lottery wins a car? (**0.1%**, 1%, 10%, 0.5%)^b^  Q1. Imagine that we roll a fair, six-sided die 1000 times. Out of 1000 rolls, how many times do you think the die would come up as an even number? (300, 167, 150, or **500)**  Q9. If the chance of getting a disease is 20 out of 100, this would be the same as having _____ chance of getting the disease. (80%, 2%, **20%**, 40%) |

*Note*. Correct answers are in boldface. The name of the items refers to the original test by Weller et al. (2013).

^a^The item was readapted to match the cultural context; the original item was about a bat and a ball.

^b^The item was readapted to match cultural context; in the original version, the lottery's name was ACME PUBLISHING SWEEPSTAKES.
