# Supplementary material for: The polarizing impact of numeracy, economic literacy, and science literacy on the perception of immigration
Source: PLoS One. 2022 Oct 7;17(10):e0274680. doi: 10.1371/journal.pone.0274680 (PMC9543957; doi:10.1371/journal.pone.0274680)
Supplement: S9 Table — Descriptive statistics for numeracy. (DOCX) [file pone.0274680.s009.docx]

**Table S9. Numeracy descriptives**. Descriptive statistics for numeracy

|  | Sample mean | Standard deviation | Median | Minimun | Maximum | Number of observations | Missing |
| --- | --- | --- | --- | --- | --- | --- | --- |
| Q16 | 0.36 | 0.48 | 0 | 0 | 1 | 551 | 0 |
| Q15 | 0.44 | 0.50 | 0 | 0 | 1 | 551 | 0 |
| Q3 | 0.84 | 0.37 | 1 | 0 | 1 | 551 | 0 |
| Q1 | 0.66 | 0.47 | 1 | 0 | 1 | 551 | 0 |
| Q9 | 0.91 | 0.28 | 1 | 0 | 1 | 551 | 0 |
| Numeracy | 3.22 | 1.11 | 3 | 0 | 5 | 551 | 0 |
