# Supplementary material for: The polarizing impact of numeracy, economic literacy, and science literacy on the perception of immigration
Source: PLoS One. 2022 Oct 7;17(10):e0274680. doi: 10.1371/journal.pone.0274680 (PMC9543957; doi:10.1371/journal.pone.0274680)
Supplement: S10 Table — Items used in the survey to measure economic literacy [46]. (DOCX) [file pone.0274680.s010.docx]

**Table S10. Economic items**. Items used in the survey to measure economic literacy (46)

| Economic Literacy |
| --- |
| ITEM 8. If the government decides to reduce the payroll taxes on the wages and salaries of workers, then there will most likely be: A. a decrease in saving. B. a decrease in investment. C. an increase in consumption. D. an increase in unemployment.  ITEM 9. A high school student buys a sweatshirt from a store. The sweatshirt is on sale at a 20 percent discount off the regular price. In this exchange, A. the student and the store benefit. B. the student benefits, but the store does not. C. the store benefits, but the student does not. D. neither the student nor the store benefits.  ITEM 13. When there is a shortage of a product in a competitive market, it is usually the case that the A. market price of the product will eventually increase. B. market price of the product will eventually decrease. C. quantity of the product exchanged in the market will eventually decrease. D. quantity of the product exchanged in the market will not change, but demand will increase.  ITEM 15. In a competitive market, the price of a product is $5.00. If the government passes a law that sets a minimum price of the product at $6.00, this change will most likely result in A. a surplus of the product. B. a shortage of the product. C. a decrease in the supply of the product. D. an increase in the demand for the product.  ITEM 17. In a competitive market, the price of wheat is likely to be increased by A. a decrease in the supply of wheat. B. a decrease in the demand for wheat. C. more capital investment in wheat farms. D. new machines reducing the cost of producing wheat.  ITEM 23. What primary function is money serving when it is used to buy a ticket to a movie? A. Store of value. B. Flow of funds. C. Unit of account. D. Medium of exchange.  ITEM 25. Inflation is an increase in A. interest rates over time. B. the standard of living over time. C. the general level of prices over time. D. real gross domestic product over time.  ITEM 26. An increase in real interest rates provides an incentive for people to save A. less and borrow less. B. more and borrow less. C. less and borrow more. D. more and borrow more.  ITEM 30. A basic role of entrepreneurs in the economy is to A. create dividends for investors in new businesses. B. buy and sell the common stocks of new corporations. C. take the risks associated with starting new businesses. D. show government what new products the economy can produce and sell.  ITEM 41. An economy will typically experience a decline in its unemployment rate when there is A. an increase in population. B. a decrease in consumer incomes. C. an increase in economic growth. D. a decrease in business investment.  ITEM 42. If your annual income rises by 50% while prices of the things you buy rise by 100%, then your A. real income has risen. B. real income has fallen. C. money income has fallen. D. real income is not affected.  ITEM 44. A government budget deficit exists when A. tax revenues are falling. B. government spending is rising. C. the national debt is decreasing. D. government spending is greater than tax revenues. |
