# Supplementary material for: The polarizing impact of numeracy, economic literacy, and science literacy on the perception of immigration
Source: PLoS One. 2022 Oct 7;17(10):e0274680. doi: 10.1371/journal.pone.0274680 (PMC9543957; doi:10.1371/journal.pone.0274680)
Supplement: S11 Table — Descriptive statistics for economic literacy. (DOCX) [file pone.0274680.s011.docx]

**Table S11. Economic descriptives**. Descriptive statistics for economic literacy

|  | Sample mean | Standard deviation | Median | Minimun | Maximum | Number of observations | Missing |
| --- | --- | --- | --- | --- | --- | --- | --- |
| ITEM 8 | 0.90 | 0.29 | 1 | 0 | 1 | 551 | 0 |
| ITEM 9 | 0.77 | 0.42 | 1 | 0 | 1 | 551 | 0 |
| ITEM 13 | 0.89 | 0.31 | 1 | 0 | 1 | 551 | 0 |
| ITEM 15 | 0.64 | 0.48 | 1 | 0 | 1 | 551 | 0 |
| ITEM 17 | 0.73 | 0.44 | 1 | 0 | 1 | 551 | 0 |
| ITEM 23 | 0.68 | 0.47 | 1 | 0 | 1 | 551 | 0 |
| ITEM 25 | 0.73 | 0.45 | 1 | 0 | 1 | 551 | 0 |
| ITEM 26 | 0.71 | 0.45 | 1 | 0 | 1 | 551 | 0 |
| ITEM 30 | 0.48 | 0.50 | 0 | 0 | 1 | 551 | 0 |
| ITEM 41 | 0.85 | 0.36 | 1 | 0 | 1 | 551 | 0 |
| ITEM 42 | 0.69 | 0.46 | 1 | 0 | 1 | 551 | 0 |
| ITEM 44 | 0.82 | 0.38 | 1 | 0 | 1 | 551 | 0 |
| Economic literacy | 8.91 | 2.67 | 9 | 0 | 12 | 551 | 0 |
