# Supplementary material for: The polarizing impact of numeracy, economic literacy, and science literacy on the perception of immigration
Source: PLoS One. 2022 Oct 7;17(10):e0274680. doi: 10.1371/journal.pone.0274680 (PMC9543957; doi:10.1371/journal.pone.0274680)
Supplement: S12 Table — Items used in the survey to measure science literacy [85]. (DOCX) [file pone.0274680.s012.docx]

**Table S12. Science Items**. Items used in the survey to measure science literacy (64)

| Science Literacy |
| --- |
| Q1.The center of the Earth is very hot.  Q2. All radioactivity is man-made.  Q3. It is the father’s gene that decides whether the baby is a boy or a girl.  Q4. Lasers work by focusing sound waves.  Q5. Electrons are smaller than atoms.  Q6. Antibiotics kill viruses as well as bacteria.  Q7. The continents on which we live have been moving their locations for millions of years and will continue to move in the future.  Q8. It is the Earth that goes around the Sun.  Q9. According to the theory of evolution, human beings, as we know them today, developed from earlier species of animals.  Q10. According to astronomers, the universe began with a big explosion. |
