# Supplementary material for: The polarizing impact of numeracy, economic literacy, and science literacy on the perception of immigration
Source: PLoS One. 2022 Oct 7;17(10):e0274680. doi: 10.1371/journal.pone.0274680 (PMC9543957; doi:10.1371/journal.pone.0274680)
Supplement: S13 Table — Descriptive statistics for science literacy. (DOCX) [file pone.0274680.s013.docx]

**Table S13. Science descriptives**. Descriptive statistics for science literacy

|  | Sample mean | Standard deviation | Median | Minimun | Maximum | Number of observations | Missing |
| --- | --- | --- | --- | --- | --- | --- | --- |
| Q1 | 0.96 | 0.20 | 1 | 0 | 1 | 551 | 0 |
| Q2 | 0.91 | 0.28 | 1 | 0 | 1 | 551 | 0 |
| Q3 | 0.63 | 0.48 | 1 | 0 | 1 | 551 | 0 |
| Q4 | 0.65 | 0.48 | 1 | 0 | 1 | 551 | 0 |
| Q5 | 0.74 | 0.44 | 1 | 0 | 1 | 551 | 0 |
| Q6 | 0.74 | 0.44 | 1 | 0 | 1 | 551 | 0 |
| Q7 | 0.96 | 0.20 | 1 | 0 | 1 | 551 | 0 |
| Q8 | 0.95 | 0.21 | 1 | 0 | 1 | 551 | 0 |
| Q9 | 0.91 | 0.29 | 1 | 0 | 1 | 551 | 0 |
| Q10 | 0.94 | 0.23 | 1 | 0 | 1 | 551 | 0 |
| Science literacy | 8.39 | 1.70 | 9 | 0 | 10 | 551 | 0 |
