# Supplementary material for: The polarizing impact of numeracy, economic literacy, and science literacy on the perception of immigration
Source: PLoS One. 2022 Oct 7;17(10):e0274680. doi: 10.1371/journal.pone.0274680 (PMC9543957; doi:10.1371/journal.pone.0274680)
Supplement: S14 Table — Descriptive statistics for total literacy. (DOCX) [file pone.0274680.s014.docx]

**Table S14. Total descriptives**. Descriptive statistics for total literacy

Total literacy

|  | Sample mean | Standard deviation | Median | Minimun | Maximum | Number of observations | Missing |
| --- | --- | --- | --- | --- | --- | --- | --- |
| Total literacy | 20.52 | 4.59 | 22 | 0 | 27 | 551 | 0 |
