# Supplementary material for: The polarizing impact of numeracy, economic literacy, and science literacy on the perception of immigration
Source: PLoS One. 2022 Oct 7;17(10):e0274680. doi: 10.1371/journal.pone.0274680 (PMC9543957; doi:10.1371/journal.pone.0274680)
Supplement: S15 Table — Pearson correlations coefficients between variables. (DOCX) [file pone.0274680.s015.docx]

# **Table S15. Correlations**. Pearson correlations coefficients between variables.

|  | 1 | 2 | 3 | 4 |
| --- | --- | --- | --- | --- |
| 1. Perception of Immigration | -- |  |  |  |
| 2. Worldviews | .477*** | -- |  |  |
| 3. Numeracy | -.048 | -.096* | -- |  |
| 4. Economic Literacy | -.102* | -.070 | .457*** | -- |
| 5. Science Literacy | -.138*** | -.161*** | .447*** | .588*** |

*Note.* *indicates *p* <.05, ** indicates *p* <.01, *** indicates *p* <.001.
