# Supplementary material for: The polarizing impact of numeracy, economic literacy, and science literacy on the perception of immigration
Source: PLoS One. 2022 Oct 7;17(10):e0274680. doi: 10.1371/journal.pone.0274680 (PMC9543957; doi:10.1371/journal.pone.0274680)
Supplement: S1 File — R scripts and explanations of the simulation with the dichotomization and non-dichotomization of the continuous moderating variable. (PDF) [file pone.0274680.s016.pdf]

Additional material to *The polarizing impact of numeracy, economic literacy, and science literacy on the perception of immigration*

In the framework of the presented simulation, we consider a quantitative variable `z` (mean of 12 items) and a binary variable `z2` that split out the cases in 2 worldview groups: *right-oriented* (when `z2=1`) and *left-oriented* (when `z2=0`). The variable `z2` has been obtained dichotomizing the variable `z` based on a *cut-off* equal to 2.5 (the value 2.5 is assigned to the *left-oriented*). In the following, it is reported the contingency table of the cases.

```
table(df1$z > 2.5,df1$z2)
```

```
##
##          0   1
##   FALSE 214   0
##   TRUE    0 334
```

The model

Consider the following model $\hat{y} = 3.15 + 0.12x - 0.15z - 0.12xz$ where z is a binary variable identifying two distinct sub-populations (groups), which we call A ($z = 1$, i.e. right-oriented) and B ($z = 0$, left-oriented). It is important to remark that these two sub-populations exist *a priori*. The model mirrors the regression model estimated in the paper on the data collected by the survey (for the model output, see the main text).

Therefore, two distinct regression lines exist:

- the first, related to the group A (right-oriented), is: $\hat{y}_A = 3$
- the second, related to the group B (left-oriented), is: $\hat{y}_B = 3.15 + 0.12x$.

Hence, in the group A (right-oriented) the correlation between x and the dependent variable y is null, while in the group B (left-oriented) the correlation is greater than zero (given the sign of the regression coefficient).

This is how basically happen in the data (although dichotomizing the quantitative variable `z`), considering as dependent variable the *numeracy*:

```
x <- df1$x
z2 <- df1$z2
y <- df1$NewY
#
xa <- x[z2 == 1]; xb <- x[z2 == 0]
ya <- y[z2 == 1]; yb <- y[z2 == 0]
cor(xa,ya)
```

```
## [1] -0.03092448
```

```
cor(xb,yb)
```

```
## [1] 0.1933798
```

The observed values y_i have, inside each group, a *systematic component* ($\hat{y}_i$) and a *random component* (e_i); the latter follows a normal distribution with mean 0 and standard deviation 0.7 (mainly the valued obtained

with our data).

The reference simulation

We generate the data, mirroring the hypothesized situation. The reference simulation is then:

```
#
s <- 0.7
nrep <- 40*6 # multiples of 6
x <- rep(c(0:5), each=nrep/6) # the covariate x ranges from 0 to 5

set.seed(654321)

# group A (right-oriented)
za <- rep(1,nrep)
ya <- rnorm(nrep,3,s)
cor(x,ya)
```

```
## [1] -0.02467126
```

```
# group B (left-oriented)
zb <- rep(0,nrep)
eb <- rnorm(nrep,0,s)
yb <- 3.15 + 0.12*x + eb
cor(x,yb)
```

```
## [1] 0.2643243
```

```
# attach the data of the two groups and fit the regression
z <- c(za,zb)
x <- c(x,x)
y <- c(ya,yb)
```

With 240 observations per group (near to the responses of our survey), the fitted values (estimates) mirror quite closely those of the hypothesized model:

```
fit <- lm(y ~ x*z); summary(fit)
```

```
##
## Call:
## lm(formula = y ~ x * z)
##
## Residuals:
##     Min      1Q  Median      3Q     Max
## -2.1167 -0.4963  0.0194  0.5145  2.4209
##
## Coefficients:
##             Estimate Std. Error t value Pr(>|t|)
## (Intercept)  3.14657    0.08383  37.535  < 2e-16 ***
## x            0.11165    0.02769   4.032 6.43e-05 ***
## z           -0.14551    0.11855  -1.227  0.22031
## x:z         -0.12266    0.03916  -3.132  0.00184 **
## ---
## Signif. codes:  0 '***' 0.001 '**' 0.01 '*' 0.05 '.' 0.1 ' ' 1
##
## Residual standard error: 0.7326 on 476 degrees of freedom
## Multiple R-squared:  0.1155, Adjusted R-squared:  0.1099
```

```
## F-statistic: 20.71 on 3 and 476 DF,  p-value: 1.255e-12
```

In the operational practice, we do not know if a unit belong to the sub-population A or to the sub-population B. We know only the numerical value of the quantitative variable (that we call u), normal distributed with mean μ_A in the group A and mean μ_B in the group B, with same standard deviation σ. We have that $\mu_A > \mu_B$ (high scores are obtained by right-oriented units); for instance, we can have $\mu_A = 3$ and $\mu_B = 2$, such that the central value is 2.5. To assign a unit to the group A (right-oriented) or to the group B (left-oriented) we observe if the u value is higher or lower than 2.5: if $u \leq 2.5$ we assign the unit to the group B (left-oriented), if $u > 2.5$ we assign the unit to group A (right-oriented).

If the two groups are well separated, the situation would not be very different than that described previously. For instance, if we had $\sigma = 0.15$ the result of the previous simulation (adapted on the basis of the values of the dichotomous quantitative variable u using the threshold 2.5) would be:

```
s <- 0.7
nrep <- 40*6 # multiples of 6
x <- rep(c(0:5), each=nrep/6) # the covariate x ranges from 0 and 5
set.seed(654321)

# group A (right-oriented)
za <- rep(1,nrep)
ya <- rnorm(nrep,3,s)
# group B (left-oriented)
zb <- rep(0,nrep)
eb <- rnorm(nrep,0,s)
yb <- 3.15 + 0.12*x + eb

# introduce the quantitative variable u
ma <- 3; mb <- 2; s  <- 0.15
ua <- rnorm(nrep,ma,s)
ub <- rnorm(nrep,mb,s)
u <- c(ua,ub)
zz <- (u > 2.5)*1
table(c(za,zb),zz)
```

```
##    zz
##       0   1
##   0 240   0
##   1   0 240
```

As can be seen, any classification error would not exist and the regression result would be, obviously, equal to the previous one:

```
x <- c(x,x)
y <- c(ya,yb)
fit <- lm(y ~ x*zz); summary(fit)
```

```
##
## Call:
## lm(formula = y ~ x * zz)
##
## Residuals:
##     Min      1Q  Median      3Q     Max
## -2.1167 -0.4963  0.0194  0.5145  2.4209
##
## Coefficients:
##             Estimate Std. Error t value Pr(>|t|)
```

```
## (Intercept)  3.14657    0.08383  37.535  < 2e-16 ***
## x             0.11165    0.02769   4.032 6.43e-05 ***
## zz           -0.14551    0.11855  -1.227  0.22031
## x:zz         -0.12266    0.03916  -3.132  0.00184 **
## ---
## Signif. codes:  0 '***' 0.001 '**' 0.01 '*' 0.05 '.' 0.1 ' ' 1
##
## Residual standard error: 0.7326 on 476 degrees of freedom
## Multiple R-squared:  0.1155, Adjusted R-squared:  0.1099
## F-statistic: 20.71 on 3 and 476 DF,  p-value: 1.255e-12
```

The regression results obtained using the quantitative variable u instead of the binary one would be more difficult to interpret, especially concerning the interpretation of the interaction among two quantitative variables. In addition, the regression coefficient of the covariate x would result almost three time (even if it is not possible a direct comparison among the two coefficients).

```
fit <- lm(y ~ x*u); summary(fit)
```

```
##
## Call:
## lm(formula = y ~ x * u)
##
## Residuals:
##      Min       1Q   Median       3Q      Max
## -2.14183 -0.53811 -0.00128  0.52482  2.44766
##
## Coefficients:
##             Estimate Std. Error t value Pr(>|t|)
## (Intercept)  3.44047    0.29383  11.709  < 2e-16 ***
## x             0.29828    0.09524   3.132  0.00184 **
## u            -0.14661    0.11485  -1.276  0.20240
## x:u          -0.09940    0.03722  -2.670  0.00784 **
## ---
## Signif. codes:  0 '***' 0.001 '**' 0.01 '*' 0.05 '.' 0.1 ' ' 1
##
## Residual standard error: 0.7393 on 476 degrees of freedom
## Multiple R-squared:  0.09902,    Adjusted R-squared:  0.09334
## F-statistic: 17.44 on 3 and 476 DF,  p-value: 9.315e-11
```

The automated simulation

We define a function that allows to see the variation of the results (especially the statistical significance of the interaction estimate) when one modifies the standard deviation of the analyzed populations:

```
job <- function(s, nrep, seed=654321) {
  x <- rep(c(0:5), each=nrep/6) # the covariate x ranges from 0 and 5
  set.seed(seed)
  # the standard deviation of the residuals is fixed to 0.7
  za <- rep(1,nrep); ya <- rnorm(nrep,3,0.7) # group A (right-oriented)
  # group B (left-oriented)
  zb <- rep(0,nrep); eb <- rnorm(nrep,0,0.7); yb <- 3.15 + 0.12*x + eb
  z <- c(za,zb); y <- c(ya,yb)
  x <- c(x,x)
  # introduce the quantitative variable u
  ma <- 3; mb <- 2
  ua <- rnorm(nrep,ma,s)
```

```r
  ub <- rnorm(nrep,mb,s)
  u <- c(ua,ub)
  zz <- (u > 2.5)*1
  out <- data.frame(y,x,z,u,zz)
}
```

The arguments of the function are:

- the standard deviations of the analyzed populations (the standard deviation of u);
- the number of replicates (must be a multiple of 6);
- the trigger for the function `rnorm`.

The function gives as output a *dataframe* whose columns have the following meaning:

- the observed values of the dependent variable (generated according to the previously defined model);
- the values of the covariate x (that are substantially fixed and equal for any simulation);
- the true identification of the group membership: $z = 1$ for right-oriented and $z = 0$ for left-oriented;
- the value of the quantitative variable u that allow identifying the group membership to group A (right-oriented) or to group B (left-oriented);
- the dichotomous value of the quantitative variable u obtained using the fixed *cutoff* equal to 2.5.

The function has, in addition, some internally fixed parameters, such as the values of μ_A and μ_B and the standard deviation of the regression model (the standard deviation of the regression model residuals).

We verify that the results are identical to the previous ones if $\sigma = 15$:

```r
df <- job(0.15, 40*6, 654321)
fit <- lm(y ~ x*zz, data=df); summary(fit)
```

```
##
## Call:
## lm(formula = y ~ x * zz, data = df)
##
## Residuals:
##     Min      1Q  Median      3Q     Max
## -2.1167 -0.4963  0.0194  0.5145  2.4209
##
## Coefficients:
##             Estimate Std. Error t value Pr(>|t|)
## (Intercept)  3.14657    0.08383  37.535  < 2e-16 ***
## x            0.11165    0.02769   4.032 6.43e-05 ***
## zz          -0.14551    0.11855  -1.227  0.22031
## x:zz        -0.12266    0.03916  -3.132  0.00184 **
## ---
## Signif. codes:  0 '***' 0.001 '**' 0.01 '*' 0.05 '.' 0.1 ' ' 1
##
## Residual standard error: 0.7326 on 476 degrees of freedom
## Multiple R-squared:  0.1155, Adjusted R-squared:  0.1099
## F-statistic: 20.71 on 3 and 476 DF,  p-value: 1.255e-12
```

We see what happen doubling the standard deviation ($\sigma = 0.3$):

```r
df <- job(0.30, 40*6, 654321)
```

Now no longer exists a perfect overlap and some (a few) classification errors appear:

```r
table(df$z,df$zz)
```

```
##
```

```
##       0   1
##   0 226  14
##   1   5 235
```

We compare the parameter estimates of the two models (that with the dichotomous variable and that with the quantitative variable u, non-dichotomous) and, in particular, the associated *p-values*:

```
fit.1 <- lm(y ~ x*zz, data=df); tmp.1 <- summary(fit.1)$coef
fit.2 <- lm(y ~ x*u, data=df);  tmp.2 <- summary(fit.2)$coef
cbind(tmp.1[,c(1,4)],tmp.2[,c(1,4)])
```

```
##                Estimate     Pr(>|t|)    Estimate     Pr(>|t|)
## (Intercept)  3.12283124 6.668959e-139  3.38212904 5.605678e-31
## x            0.10924741  1.367090e-04  0.23365964 7.476675e-03
## zz          -0.09015219  4.545095e-01 -0.12320103 2.437956e-01
## x:zz        -0.11523979  3.937171e-03 -0.07368056 2.964237e-02
```

The interaction is statistically significant for both models (but the *p-value* for the first model is lower than that obtained for the second, i.e. most significant).

We increase again the standard deviation, reaching up to $\sigma = 0.4$:

```
df <- job(0.40, 40*6, 654321)
```

The classification errors increase:

```
table(df$z,df$zz)
```

```
##
##       0   1
##   0 219  21
##   1  20 220
```

Comparing the parameter estimates of the models (the first with the dichotomous variable and the second with the non-dichotomous variable), we observe now that, while for the first the interaction is still significant, the second shows a non significant value for the interaction (even if quite near to the significance limit):

```
fit.1 <- lm(y ~ x*zz, data=df); tmp.1 <- summary(fit.1)$coef
fit.2 <- lm(y ~ x*u, data=df);  tmp.2 <- summary(fit.2)$coef
cbind(tmp.1[,c(1,4)],tmp.2[,c(1,4)])
```

```
##               Estimate     Pr(>|t|)   Estimate     Pr(>|t|)
## (Intercept)  3.1003082 1.379425e-136  3.3313012 7.371101e-34
## x            0.1062085  2.269657e-04  0.1979778 1.462091e-02
## zz          -0.0525424  6.652281e-01 -0.1028468 2.942627e-01
## x:zz        -0.1124319  5.231866e-03 -0.0594416 5.728440e-02
```

If we increase again the standard deviation, reaching up to $\sigma = 0.5$:

```
df <- job(0.50, 40*6, 654321)
```

It is confirmed a statistically significant interaction for the model with the dichotomous variable (even if with a *p-value* lower than that observed in the previous simulation), while the model that considers the non-dichotomous variable shows, concerning the interaction estimate, a value definitely not significant:

```
fit.1 <- lm(y ~ x*zz, data=df); tmp.1 <- summary(fit.1)$coef
fit.2 <- lm(y ~ x*u, data=df);  tmp.2 <- summary(fit.2)$coef
cbind(tmp.1[,c(1,4)],tmp.2[,c(1,4)])
```

```
##                Estimate     Pr(>|t|)    Estimate     Pr(>|t|)
## (Intercept)  3.11300284 7.583713e-136  3.28317496 2.132925e-37
```

```
## x             0.09777111  7.043476e-04  0.16947536 2.378296e-02
## zz           -0.07299883  5.495359e-01 -0.08359244 3.545228e-01
## x:zz         -0.09824388  1.502084e-02 -0.04804583 9.421730e-02
```

Analogous result (more evident) if we set $\sigma = 0.6$:

```
df <- job(0.60, 40*6, 654321)
fit.1 <- lm(y ~ x*zz, data=df); tmp.1 <- summary(fit.1)$coef
fit.2 <- lm(y ~ x*u, data=df);  tmp.2 <- summary(fit.2)$coef
cbind(tmp.1[,c(1,4)],tmp.2[,c(1,4)])
```

```
##                Estimate      Pr(>|t|)    Estimate     Pr(>|t|)
## (Intercept)  3.07070684 8.240613e-133  3.24202679 1.696461e-41
## x            0.09573929  8.962084e-04  0.14720042 3.368570e-02
## zz           0.01257222  9.187594e-01 -0.06714215 4.179731e-01
## x:zz        -0.09720912  1.732779e-02 -0.03912399 1.372890e-01
```

We observed that, in presence of classification errors, it is preferable to implement a model with the dichotomous variable than that with numerical variable (continuous). In fact, the test on the interaction used in the first case is more powerful than that used in the second.
