# Supplementary material for: The polarizing impact of numeracy, economic literacy, and science literacy on the perception of immigration
Source: PLoS One. 2022 Oct 7;17(10):e0274680. doi: 10.1371/journal.pone.0274680 (PMC9543957; doi:10.1371/journal.pone.0274680)
Supplement: Attachment — Submitted filename: Response to Reviewers.docx [file pone.0274680.s017.docx]

**RESPONSE TO REVIEWERS**

**RESPONSE TO REVIEWER 1**

**Dear Reviewer #1,**

**we thank you for your careful reading of the manuscript and the useful advices you gave us. We have accepted all of your suggestions except for one point (point #4 concerning the dichotomization of the continuous variable and point #9 and #10). The reasons for our resistance are detailed below. We believe that the manuscript is now much improved with the changes we have made, and we are grateful to you for this.**

1. Please indicate how duplicate responses were prevented in the online survey (e.g., unique survey links, I.P. addresses, etc.).

**ANSWER: Thank you for noting this. Each participant was sent a unique (and anonymous) code identifier by mail with which they could access the online questionnaire. The code could be used only once. We clarified this in the manuscript adding a couple of sentences as follows:**

***"*** ***Each participant was sent a unique (and anonymous) code identifier by mail with which they could access the online questionnaire. A single usage of the code was allowed. No signed informed consent was collected, but the participant gave electronic informed consent by accessing the questionnaire with their unique code and agreeing to complete it online. The participant needing help in compiling the form contacted the telephone number and provided their unique code to the experimenter, who accessed the questionnaire on their behalf and read the questions to the participant by phone and completed the questionnaire***. ***"***

2. Was the survey conducted in Italian or English?

**ANSWER: We apologize for the lack of clarity. The survey was conducted in Italian. We have now clarified this point in the method section of the manuscript. We added a sentence:**

***"*** ***The questionnaire was in Italian, the respondents' native language."***

3. Please provide a rationale for choosing the subset of economic literacy questions.

**ANSWER: Thank you for allowing us to clarify this point. The original Test of Economic Literacy by Walstad, Rebeck, and Butters (2013) includes 45 items covering 20 content standards. For a survey like ours that investigated multiple constructs (5 macro constructs) on large numbers of individuals (a representative sample of the city population), 45 items would have been too many.** **As there is no short form of the test, we selected a smaller number of 12 items according to two criteria: (i) the items had to be simple enough not to elicit a refusal response from respondents (volunteers) driven by the feeling of being under scrutiny; (ii) the items had to be distributed across different content standards so as to be representative of the different contents of the test.** **Therefore, we used the U.S. data on item difficulty to select a subset of items that were both easy (over 40% correct responses in the student sample not enrolled in a basic course with economics) and representative of different contents.** **The 12 selected items had a difficulty ranging from a minimum of 42.3% to a maximum of 66.3% correct responses, with an average of 51% correct responses.** **In addition, the items investigated the following 9 contents: (a) Economic incentives - prices, wages, profits, etc. (item 8); (b) Voluntary exchange and trade (item 9); (c) Markets and prices (item 13); (d) Supply and demand (items 15 and 17); (e) Money and inflation (items 23 and 25); (f) Interest rates (item 26); (g) Entrepreneurship (item 30); (h) Unemployment and inflation (item 41 and item 42); (i) Fiscal and monetary policy (item 44).**

**We have added this information in the manuscript in the section on "Economic Literacy":**

***" Items were selected to meet two criteria: (1) they had to be sufficiently easy (more than 40% correct responses in the U.S. student sample not enrolled in a basic course with economics, as reported in*** (Walstad et al., 2013)***); (2) they had to be representative of different test contents. The subset of 12 items selected had a difficulty that ranged from a minimum of 42.3% to a maximum of 66.3% correct responses, with an average of 51% correct responses. In addition, the items investigated the following 9 contents out of 20: (a) Economic incentives - prices, wages, profits, etc (item 8); (b) Voluntary exchange and trade (item 9); (c) Markets and prices (item 13); (d) Supply and demand (items 15 and 17); (e) Money and inflation (items 23 and 25); (f) Interest rates (item 26); (g) Entrepreneurship (item 30); (h) Unemployment and inflation (items 41 and item 42); (i) Fiscal and monetary policy (item 44). Individual score on economic literacy was computed by calculating the number of correct answers (M = 8.91; SD = 2.67).***

4. Dichotomization of quantitative variables is not ideal and should only be used in very specific, theoretically-derived conditions (MacCallum et al., 2002, citation below). The authors say that this is not an issue because results were similar but don't provide the results for verification. The fact that another article used a non-ideal analysis isn't sufficient justification. Please conduct the analyses using the full ideological scales. I suggest mean-centering your quantitative variables involved in interactions to aid in interpretation of any main effects.

**ANSWER: Thank you for pointing this out. The article by MacCallum et al. (2002) is a very interesting read. The arguments of MacCallum et al. (2002) are certainly right and of great importance. Indeed, the article shows the risks of dichotomization in the presence of continuous reality. And on this assumption, is based. As well as the simulations that are proposed, which start from a continuous reality. Our study's situation is quite different.** **We strongly believe that our reality is made up of two groups, with a certain degree of overlap. Of course, with the possibility of making classification errors. The scale used in the questionnaire is conceived to identify them. In fact, the items measuring the cultural worldview are aimed at producing a dichotomization. Our paper assumes that there are two pre-existing and predefined subpopulations and that the variable we have used (worldview orientation) helps us to identify them. Following MacCallum et al. (2002), we performed a simulation that demonstrates how using the continuous variable to predict a dichotomized reality reduces the power of the interaction test (which is the parameter of our primary interest). We show some of the most relevant results that emerged from that simulation in the attached file (see S1 File). Because the reader might legitimately have the same concerns as you did, we have added this sentence in the manuscript in the section on data analysis and made available the simulation in supplementary materials.**

***"*** ***This dichotomization does not constitute a forcing nor an excessive simplification and, especially, does not introduce any distortion in the proposed and estimated models, such as significative interactions which would otherwise not exist. Dichotomization can yield misleading results in the presence of continuous reality (MacCallum et al., 2002). However, we believe that our reality is made up of two groups with a certain degree of overlap. The cultural worldviews scale is conceived to identify them, of course, with the possibility of making classification errors. The worldview orientation scale uses a continuous measurement for research purposes, i.e., the need to elicit truthful answers to sensitive ideological questions. However, the items measuring cultural worldviews are aimed at producing a dichotomization, i.e., a classification of an individual as hierarchical-individualistic or egalitarian-communitarian. Following MacCallum et al. (2002), we performed a simulation that demonstrates how using the continuous variable to predict a dichotomized reality reduces the power of the interaction test (which is the parameter of our primary interest). We show some of the most relevant results that emerged from that simulation in the attached file (see S1 File).***

5. "the parameter associated to the non-binary variable (say ̅ ) resulted however significantly different from zero" is unclear. Please clarify what this means.

**ANSWER: Thank you for noticing it. We have deleted this sentence which was definitely not clear. Moreover, it is now unnecessary, given the specifications that we included relative to the dichotomization at the previous point (#4).**

6. Table 1 refers to x, y, and z and betas with subscripts. Please indicate which variable each term represents rather than forcing the reader to refer back to the regression equation. I think, but am not sure, that b3 was the interaction, but then page 19 refers to the b3 row for the non-significant effects of worldview. Indicating the effects using words would eliminate the potential for confusion about which coefficient corresponds to which tested effect.

**ANSWER: Thank you for the careful reading of the results and Table 1. There was a mistake: b_3_ should have been b_2_; we have now corrected it. Moreover, as suggested, we made explicit the variable to which lines b_0_, b_1_, b_2,_ and b_3_ in Table 1 refer: the intercept, the literacy variable, the worldviews, and the interaction, respectively.**

7. Showing polarization with respect to social issues is not novel. Political scientists have long known (e.g., Converse, 2000) that more knowledgeable people are more polarized in their political attitudes. In addition, Ganzach (2018) showed that multiple forms of intelligence predict polarization in political attitudes. It is therefore unclear whether your findings are due to your hypothesized rationale for those variables specifically being related to polarization or whether your findings are due to intelligence, in general.

**ANSWER: Thank you for suggesting this alternative interpretation of our findings based on the work of Converse (2000) and Ganzach (2018). Both papers fit with our study, so we decided to include them in the discussion to address the issue of whether our findings are due to intelligence and not to the knowledge and ability variables we have measured. The topic is really interesting, and after several considerations, we concluded that it is of course, possible to relate increased knowledge/ability with intelligence. This evidence might raise the question of whether our results are due to the specific variables we have examined (i.e., numerical, economic, and science literacy) or, rather, to intelligence. However, at least for numeracy, there are data that indicate that numeracy explains behavior even when controlling for intelligence.** **For example, the positive relationship between numeracy and comprehension of numerical data remains even after controlling for measures of intelligence** (Låg et al., 2014)**. Seemingly, lower objective numeracy has been associated with more conjunction errors, even after controlling for intelligence measures** (Liberali et al., 2012)**. Numerical ability and intelligence are therefore related, but they are not completely overlapping. We do not know as regards the other two constructs. Intelligence, therefore, may certainly be part of the explanation, but to rule out the explanation, one should measure intelligence. This issue, indeed, is a perfect ground for further studies. Strongly inspired by your comments, we have added a paragraph about this in the conclusions:**

***"Some research, however, has raised concerns about the robustness of the explanation based on cultural cognition*** (Persson et al., 2021)***. The fact that higher knowledge is associated with greater cultural polarization of concern about immigration is consistent with the motivated reasoning explanation, yet, other explanations may also apply. For example, numeracy effects may actually be due to variance shared with other types of intelligence, such as verbal ability in solving analogies*** (Shoots-Reinhard et al., 2021)***. It is known, indeed, that more informed individuals are usually more polarized in their political attitudes*** (e.g., Converse, 2000) ***and that multiple forms of intelligence can predict the polarization of political attitudes*** (Ganzach, 2018)***. This evidence might raise the question of whether our results are due to the specific variables we have examined (i.e., numerical, economic, and science literacy) or, rather, to intelligence. As regards numeracy, it has been shown that individual behavior can be explained by numeracy, even after controlling for intelligence. For example, the positive relationship between numeracy and comprehension of numerical data persists even after controlling for measures of intelligence*** (Låg et al., 2014)***. Seemingly, conjunction errors are predicted by lower objective numeracy, even after controlling for intelligence measures*** (Liberali et al., 2012)***. Numeracy and intelligence are certainly strongly related, though they are not perfectly overlapping. On the other side, recent research on the polarization of COVID-19 risk perception showed that numeracy failed to predict polarization when verbal ability was also measured, suggesting that what might seem an effect of numeracy is indeed an effect of cognitive ability, such as verbal ability (30). Subsequent studies should measure individual intelligence as well as individual knowledge to directly compare the respective predictive effect and address the question of whether polarized views of immigration are better explained by intelligence rather than knowledge or education”.***

8. Your discussion that your use of cultural worldviews being necessary because the political landscape in Italy cannot be reduced to unidimensional left-right classification, yet you reduced your measure of ideology to two dimensions and then further conducted a median split.

**ANSWER: You are completely right. We were definitely not very logical in our explanations, as you rightly pointed out, and we are sorry to have caused confusion. We have now clarified that what we meant when we said that the political landscape in Italy is complex and that a two-dimensional measure of political orientation was not appropriate** **was that the typical measure used for political orientation would not apply to our context. To be more clear, few Italians would be able to tell whether they are more liberal or conservative, there are no bipolar-parties such as republicans and democrats and few would be able to classify themselves as clearly right or left. Perhaps Italy is going through a period of socio-political transformation because we observe that many who used to be left-wingers now vote right-wing not because they have become right-wingers but because they believe that the battles of the left are conducted more by right-wing politicians than by left-wing ones. And vice versa. To a straightforward question, such as "are you right-wing or left-wing" these people would not know what to answer because they are left-wing but vote to the right. We believed instead that a more detailed and nuanced measure of the underlying ideology of individual beliefs and behaviors, such as worldviews, would better serve the purpose of dichotomizing the sample into two polarized groups. We justified this decision in the following way:**

***"*** ***Many studies have used a measure of political orientation to elicit the knowledge-related polarization effect*** (Drummond & Fischhoff, 2017; Hamilton et al., 2015; Smith et al., 2017)***. While in some cases this has been a pre-designed choice*** (e.g., Shoots-Reinhard et al., 2021)***, in other cases such as large-scale representative surveys, it has been an ex-post forced choice due to its availability*** (e.g., Drummond & Fischhoff, 2017)***. In deciding the ideological measure to use in our study we decided to avoid using political orientation. Political orientation is typically elicited by asking respondents to classify themselves on some bipolar dimension, such as, republican vs. democrat or liberal vs. conservative. Instead, we preferred to use a measure of cultural worldviews*** (Kahan et al., 2011)***. The reason for this choice was twofold. On the one hand, a standard question about political orientation (right-wing or left-wing) would not adapt well to our context. Indeed, the Italian political landscape is characterized by small and fragmented parties with transversal positions with respect to the standard right-wing or left-wing dichotomy. For example, the 5 Star Movement is a populist party difficult to classify as right or left*** (Roccato et al., 2020; Verbeek & Zaslove, 2016)***. It has both right-wing (e.g., anti-immigrant) and left-wing (e.g., guaranteed minimum income) ideologies, as well as both conservative (e.g., NO TAV movement) and liberal (e.g., drug liberalization) ideologies*** (Roccato et al., 2020)***. A second and more important reason is that we believe worldviews are a more detailed and nuanced measure of the underlying ideology of individual beliefs and behaviors than political orientation, with whom they do not fully overlap. Worldviews capture where an individual stands on the spectrum anchored by hierarchical-individualistic beliefs at one pole and egalitarian-communitarian beliefs at the other. They have proved to be successful in identifying a knowledge-related polarizing effect in the case of previous risk perception studies (Kahan 2912). Moreover, worldview orientations have been shown to be significant predictors of an individual’s attitudes and behaviors in the face of threats*** (Chen et al., 2020; Siegrist & Bearth, 2021; Xue et al., 2014)***, sometimes even more than political orientations*** (Dryhurst et al., 2020)***. Our study confirms that cultural worldviews may be a valid construct for measuring knowledge-related polarization effects of risk perceptions of social problems.***

9. Furthermore, the Kahan measure has two scales that could be analyzed orthogonally. If it is really the case that the worldviews in Italy are more complex, doesn't that argue for analyzing the two scales separately? Please clarify.

**ANSWER: You are correct that Kahan's scale contains two dimensions, each bipolar, whereas we have combined the questions into one dimension (hierarchical-individualistic vs. egalitarian-communitarian). We did not analyze the two scales separately because our purpose was to rank each individual on the basis of a dichotomous dimension, as explained above in point #8. Keeping the two scales separate would have complicated the reading of the results considerably: all results would have been duplicated by two and wouldn't have added much information because the two scales behave quite similarly.**

10. Your analyses do not support your conclusion that your data "show that cultural worldviews may be an ideal substitute for measuring polarization effects in areas where a two-dimensional measure of political orientation might not be as appropriate." because you reduced your measure to one dimension. Where I think your research could be novel is by showing polarization with the worldview scale in a non-US sample, assuming you do show polarization when worldviews are analyzed as continuous measures. It would be interesting and novel if you were able to show polarization is driven by hierarchical/egalitarian or individualism/communitarian subscales.

**ANSWER: Thank you for the suggestion. Keeping the two scales separate would have complicated the reading of the results considerably: all results would have been duplicated by two and wouldn't have added much information because the two scales behave quite similarly. As mentioned in 8 and 9 we made it clear that our purpose was to assign each individual to a pole of only one dichotomous dimension to show a polarization effect.**

11. Very recent research has raised some concerns about the robustness of cultural cognition findings (Persson et al., 2021) or suggested that numeracy effects are due to shared variance with other types of intelligence (Shoots-Reinhard et al., 2021). Given the present approach is strongly dependent on the past findings, some discussion of these other findings seems warranted. These two articles should be addressed in the conclusion at a minimum.

**ANSWER: Thank you for directing us to this interesting and very recent publication that we had become aware of but did not manage to include in the first version of the manuscript, which we have done in this revised version. The work by Persson and colleagues is impressive: it is a pre-registered replication of earlier work by Kahan et al 2017 on politically-consistent motivated reasoning. The replication shows that the effect is virtually absent. Although we do not cite Kahan and colleagues 2017 in our manuscript, but it is true that we do refer to "motivated reasoning", pointing to it as a possible explanation for the polarization. In doing so, we aligned ourselves with the literature suggesting this as the explanation. Our personal belief is that motivated reasoning may be one explanation but that there may be other explanations as well. For example, right-wingers might hold value schemas more rigid and unshapeable, thus also resistant to education and acquired knowledge, while left-wingers less so. This might explain why those on the left and right diverge more from each other as their knowledge increases. However, neither our study nor other classic studies of polarization have found conclusive evidence for an explanation of the phenomenon, which remains largely unexplained. The only study that went a bit deep on explanations was the study by Shoots-Reinhard et al., 2021, which suggests that polarization is induced by selective exposure and selective interpretation of information consistent with one's ideology. In the manuscript, we have softened the strength of the explanation calling into question "motivated reasoning", and expanded the discussion a bit, which now reads like this:**

***"Some research, however, has raised concerns about the robustness of the explanation based on cultural cognition*** (Persson et al., 2021)***. The fact that higher knowledge is associated with greater cultural polarization of concern about immigration is consistent with the motivated reasoning explanation, yet, other explanations may also apply. For example, numeracy effects may actually be due to variance shared with other types of intelligence, such as verbal ability in solving analogies*** (Shoots-Reinhard et al., 2021)***..".***

12. Please provide descriptive statistics (e.g., range, mean, standard deviations) for all variables. A correlation table would also be helpful.

**ANSWER: Thank you, we had forgotten to include this information in the manuscript and supplementary materials. We have now included the summary descriptive statistics (i.e., M and SD) in the method section, in the paragraph devoted to each variable. We also included the extended descriptive statistics (mean, range, standard deviation) for each question and the mean construct in dedicated tables in the supplementary materials (Tables S4-S14). Finally, we made the correlation table (Table S15) available as supplementary material as well.**

Here are full references for citations above:

MacCallum, R. C., Zhang, S., Preacher, K. J., & Rucker, D. D. (2002). On the practice of dichotomization of quantitative variables. Psychological Methods, 7(1), 19-40. doi: 10.1037/1082-989x.7.1.19

Converse, P. E. (2000). Assessing the capacity of mass electorates. Annual Review of Political Science, 3(1), 331. doi: 10.1146/annurev.polisci.3.1.331

Ganzach, Y. (2018). Intelligence and the rationality of political preferences. Intelligence, 69, 59–70. <https://doi.org/10.1016/j.intell.2018.05.002>.

Persson, E., Andersson, D., Koppel, L., Västfjäll, D., & Tinghög, G. (2021). A preregistered replication of motivated numeracy. Cognition, 214, 104768. doi: <https://doi.org/10.1016/j.cognition.2021.104768>

Shoots-Reinhard, B., Goodwin, R., Bjälkebring, P., Markowitz, D. M., Silverstein, M. C., & Peters, E. (2021). Ability-related political polarization in the COVID-19 pandemic. Intelligence, 88, 101580. doi: https://doi.org/10.1016/j.intell.2021.101580

Minor comments

13. Bruine de Bruin et al. 2020 don't report an interaction in their study; only main effects of ideology and media. However, Shoots-Reinhard et al. do show the funnel pattern for COVID, support for Medicare for all, and for weapons buyback programs. Please cite Shoots-Reinhard or another paper that documented an interaction or remove the COVID mention in the introduction.

**ANSWER: we really appreciate you noting that. Actually, you are right, Bruin de Bruin 2020 talks about polarization but does not look for interaction with education. So we removed the reference and replaced it with the one you suggested, which is much more appropriate. We still kept the Bruin de Bruin 2020 quote but moved it a bit earlier, in the paragraph where we talk about how cultural worldviews explain risk judgments of individuals in the face of threats.**

14. Missing a citation for definition of financial literacy (page 8). Perhaps Lusardi & Mitchell (2008, American Economic Review) could be used?

**ANSWER: you are absolutely right. We were so focused on economic literacy that we forgot about financial literacy. Yes, Lusardi's work is perfect. We have included it. Thank you very much.**

15. Please report only two decimal places to make it easier to parse especially tables.

**ANSWER: We agree. We reduced the decimals in the tables and rechecked all the calculations. We realized that we had forgotten to reverse code an item in the immigration perception measure, which we have now done. As a consequence, the decimals in Table 1 have changed slightly.**

16. What is the purpose of Table 2? Isn't it redundant with Figure 2?

**ANSWER: Yes indeed, we have eliminated Table 2.**

17. The Roccato citation is repeated on page 23.

**ANSWER: Yes. There was something wrong with my Mendeley, which kept repeating this entry. We have now fixed it. Thank you.**

Lusardi, A., & Mitchell, O. S. (2008). Planning and Financial Literacy: How Do Women Fare? American Economic Review, 98(2), 413-417. doi: 10.1257/aer.98.2.413

**RESPONSE TO REVIEWER 2**

**Dear Reviewer #2,**

**Thank you for your mindful reading of the manuscript and for your suggestions. We have implemented all of the recommendations you made except for # 2 and #5. Point # 2 was about using the weighted mean and not the arithmetic mean for the dependent variable perception of immigration. This suggestion, which is normally valid, turned out to be unnecessary in this particular case. We have justified our choice in detail and stand ready to modify the analyses if you deem it necessary. As for point #5 we also explained our reasons below, but we are open in case you prefer to include the results in the manuscript or in the Supporting information. All other suggestions have been implemented, and because of this, we think the manuscript is much improved. Thanks.**

1. The study investigated whether individuals' numerical ability, scientific and economic literacy impact their perception of immigration, taking into account their cultural worldview. The PRI survey used a questionnaire with standardized and ad hoc questions. A representative sample of 551 citizens of a town in the northeast of Italy, thus not a national sample. I found the paper interesting and I think the practical implications that can be derived are very important. Although it was a fairly smooth read, I think there are aspects to consider to improve its quality.

**ANSWER: We're glad you found the work interesting.**

2. - I think that the variable Perception of Immigration could have been computed more accurately, considering the weight of every single item. It would be advisable to report Cronbach's alpha index in addition to proceeding with factor analysis.

**ANSWER: Sorry for not being able to communicate these things clearly enough in the manuscript. Actually, Cronbach's alpha was calculated (**α  **= .94), but it is now reported in the paper more clearly. Also, the way the dependent variable 'perception of immigration' was constructed has been made clearer. We explained that the immigration perception variable is the simple arithmetic mean (individual by individual) of the scores given by each respondent to 13 opinion questions about immigrants. We clarified that, before performing the calculation of the averages, the scales of some variables were reversed so that higher scores correspond to a more "favorable" position on immigration. More importantly, a PCA was performed as you suggested but we feel it is not necessary to report this analysis in the main manuscript (although we make it available in the SI) and would prefer to use the mean and not the weighted mean, for three reasons:**

**(1) the arithmetic mean results in less loss of information and, therefore, greater "recovery" of data; in fact, PCA uses complete data and any partial non-response imposes exclusion from the calculation of the record for the individual who did not answer one or more questions. Below are the results of PCA, which we would prefer not to report in the manuscript, not without first showing the correlation matrices constructed by calculating Sperman's** *ρ* **(if we are to consider the nature of the data, which are to all ranks) and Pearson's linear correlation coefficients r.**

**The correlation matrix R calculated with Sperman's *ρ*:**

```
              immimp   immcult  immcrime  immiteco   immjobs    immcit
immimp     1.0000000 0.5772242 0.6368190 0.7268371 0.6024814 0.5971871
immcult    0.5772242 1.0000000 0.5940080 0.5039950 0.6209019 0.5427874
immcrime   0.6368190 0.5940080 1.0000000 0.5832409 0.6305193 0.5013359
immiteco   0.7268371 0.5039950 0.5832409 1.0000000 0.5834355 0.5881605
immjobs    0.6024814 0.6209019 0.6305193 0.5834355 1.0000000 0.5191298
immcit     0.5971871 0.5427874 0.5013359 0.5881605 0.5191298 1.0000000
immrghts   0.5218624 0.4555000 0.4305476 0.4794168 0.4471456 0.5978142
immcosts   0.6826779 0.6153745 0.6486136 0.6664441 0.6713394 0.5789071
immref     0.4902701 0.4315646 0.4222825 0.5064044 0.4882544 0.4987802
immnum     0.7099802 0.6105259 0.6834254 0.6813957 0.6220203 0.6008854
riskperc   0.7259665 0.6371458 0.7201841 0.6574490 0.6508899 0.6146545
riskaffect 0.4808500 0.4023264 0.4213511 0.4814193 0.3855928 0.4001193
riskben    0.7603968 0.5772596 0.6440378 0.7436093 0.6626731 0.6011694
            immrghts  immcosts    immref    immnum  riskperc riskaffect
immimp     0.5218624 0.6826779 0.4902701 0.7099802 0.7259665 0.4808500
immcult    0.4555000 0.6153745 0.4315646 0.6105259 0.6371458 0.4023264
immcrime   0.4305476 0.6486136 0.4222825 0.6834254 0.7201841 0.4213511
immiteco   0.4794168 0.6664441 0.5064044 0.6813957 0.6574490 0.4814193
immjobs    0.4471456 0.6713394 0.4882544 0.6220203 0.6508899 0.3855928
immcit     0.5978142 0.5789071 0.4987802 0.6008854 0.6146545 0.4001193
immrghts   1.0000000 0.4730686 0.3918708 0.5148810 0.5643132 0.3273390
immcosts   0.4730686 1.0000000 0.5890304 0.7571954 0.7112919 0.4596726
immref     0.3918708 0.5890304 1.0000000 0.5685539 0.5384318 0.2919514
immnum     0.5148810 0.7571954 0.5685539 1.0000000 0.7365787 0.4700815
riskperc   0.5643132 0.7112919 0.5384318 0.7365787 1.0000000 0.5529421
riskaffect 0.3273390 0.4596726 0.2919514 0.4700815 0.5529421 1.0000000
riskben    0.5619813 0.7192198 0.5029110 0.7575481 0.7387274 0.4925383
             riskben
immimp     0.7603968
immcult    0.5772596
immcrime   0.6440378
immiteco   0.7436093
immjobs    0.6626731
immcit     0.6011694
immrghts   0.5619813
immcosts   0.7192198
immref     0.5029110
immnum     0.7575481
riskperc   0.7387274
riskaffect 0.4925383
riskben    1.0000000
```

**The correlation matrix R constructed by calculating Pearson's linear correlation coefficients r:**

```
              immimp   immcult  immcrime  immiteco   immjobs    immcit
immimp     1.0000000 0.5997608 0.6138052 0.7455894 0.6060955 0.5878850
immcult    0.5997608 1.0000000 0.5772389 0.5048826 0.5875181 0.5324282
immcrime   0.6138052 0.5772389 1.0000000 0.5604829 0.6078344 0.4759312
immiteco   0.7455894 0.5048826 0.5604829 1.0000000 0.5839685 0.5703349
immjobs    0.6060955 0.5875181 0.6078344 0.5839685 1.0000000 0.4683891
immcit     0.5878850 0.5324282 0.4759312 0.5703349 0.4683891 1.0000000
immrghts   0.5254873 0.4558376 0.4104501 0.4737751 0.4135153 0.5851733
immcosts   0.6828013 0.6137007 0.6481052 0.6593644 0.6518089 0.5528748
immref     0.5058409 0.4006633 0.4036455 0.5120538 0.4515537 0.4636040
immnum     0.7043481 0.6003306 0.6736334 0.6625200 0.5988081 0.5848215
riskperc   0.7306793 0.6395683 0.6815122 0.6757126 0.6190803 0.6207054
riskaffect 0.4968532 0.4258549 0.4206749 0.4995838 0.3913780 0.4284992
riskben    0.7556542 0.5689027 0.6337376 0.7327885 0.6406038 0.5858643
            immrghts  immcosts    immref    immnum  riskperc riskaffect
immimp     0.5254873 0.6828013 0.5058409 0.7043481 0.7306793 0.4968532
immcult    0.4558376 0.6137007 0.4006633 0.6003306 0.6395683 0.4258549
immcrime   0.4104501 0.6481052 0.4036455 0.6736334 0.6815122 0.4206749
immiteco   0.4737751 0.6593644 0.5120538 0.6625200 0.6757126 0.4995838
immjobs    0.4135153 0.6518089 0.4515537 0.5988081 0.6190803 0.3913780
immcit     0.5851733 0.5528748 0.4636040 0.5848215 0.6207054 0.4284992
immrghts   1.0000000 0.4522670 0.3732079 0.4948080 0.5536078 0.3433215
immcosts   0.4522670 1.0000000 0.5738581 0.7577632 0.6984142 0.4687585
immref     0.3732079 0.5738581 1.0000000 0.5511433 0.5516489 0.3158073
immnum     0.4948080 0.7577632 0.5511433 1.0000000 0.7137501 0.4759196
riskperc   0.5536078 0.6984142 0.5516489 0.7137501 1.0000000 0.5832320
riskaffect 0.3433215 0.4687585 0.3158073 0.4759196 0.5832320 1.0000000
riskben    0.5455191 0.7087311 0.4995647 0.7448280 0.7265088 0.5085346
             riskben
immimp     0.7556542
immcult    0.5689027
immcrime   0.6337376
immiteco   0.7327885
immjobs    0.6406038
immcit     0.5858643
immrghts   0.5455191
immcosts   0.7087311
immref     0.4995647
immnum     0.7448280
riskperc   0.7265088
riskaffect 0.5085346
riskben    1.0000000
```

**As can be seen, the two matrices are practically superimposable.**

**Principal component analysis, performed on the 13 variables that collectively define Perception of Immigration, yielded the following results in terms of explained variability:**

```
 [1] 0.60513179 0.05942589 0.05473935 0.05043378 0.04071987 0.03288394
 [7] 0.03177319 0.02850099 0.02541370 0.01954264 0.01817199 0.01711045
[13] 0.01615243
```

**The first principal component explains about 60% of the total variability. The subsequent ones explain a proportion of total variability between 6.4% (the second) and 1.3% (the thirteenth). If one considers it more informative, one can look at the following table. The previous one is calculated, so to speak, "by hand." What follows is the output of R's prcomp function:**

```
Importance of components:
                          PC1     PC2     PC3     PC4     PC5    PC6     PC7
Standard deviation     3.3731 1.09968 1.05853 0.97019 0.85919 0.7988 0.79015
Proportion of Variance 0.6009 0.06387 0.05917 0.04971 0.03899 0.0337 0.03297
Cumulative Proportion  0.6009 0.66475 0.72393 0.77364 0.81263 0.8463 0.87930
                           PC8     PC9    PC10    PC11    PC12    PC13
Standard deviation     0.73962 0.71282 0.62653 0.57059 0.52089 0.49083
Proportion of Variance 0.02889 0.02683 0.02073 0.01719 0.01433 0.01272
Cumulative Proportion  0.90819 0.93502 0.95575 0.97295 0.98728 1.00000
```

**(2) The weights to be attributed to each individual item (the so-called factor loadings of the PCA) are substantially equal. They assume values from 0.22 to 0.35, as can be seen from the table below:**

```
    immimp    immcult   immcrime   immiteco    immjobs     immcit   immrghts
 0.2963916  0.2750613  0.2928099  0.2747562  0.2441089  0.2908017  0.2326617
  immcosts     immref     immnum   riskperc riskaffect    riskben
 0.3514382  0.2210933  0.3281109  0.2740960  0.2384599  0.2548310
```

**(3) The average of the 13 variables we have calculated shows a very high correlation with the score given by the first main component (the so-called scores, i.e. the values of the new variable defined, precisely, by the first main component). This correlation, measured with Pearson's linear correlation coefficient r, is equal to 0.98 approximately. Below we also report the scatter of our dependent variable Y against the scores attributed by the first principal component:**

**In conclusion, we feel that this evidence supports the choice of using the arithmetic mean of the 13 questions to construct the dependent variable perception of immigration, which is more intuitive and more "informative" than the first main component. Summing, and then averaging, the answers given by the respondents to the 13 questions that contribute to the construction of the variable Y is a good strategy, also because of the "I do not know/do not answer". In the manuscript we have added, in this regard, a clarification in the method section and made the statistics available in the supplementary materials (Tables S6 and S7):**

***"*** ***To be conservative, the 13 items were subjected to a principal component analysis which showed that the principal component explains about 60% of the total variability and that the weights of the items (the factor loadings of the PCA) are substantially equal to each other. In addition, the arithmetic mean of the 13 items has a very high correlation index (r = .98) with the score given by the first principal component (the scores, i.e., the values of the new variable defined, precisely, by the first principal component). We, therefore, considered it more informative to use the simple arithmetic mean as the dependent variable and not a more complex statistic, such as the mean of the items each weighted by the principal component, also because the arithmetic mean entails a lesser loss of information due to missing cases."***

3. - If I understand Table 1 correctly, the models are represented by a column. However, I found Table 1 to be unintuitive and hard to read. I suggest replacing the indices b0, b1, etc with the predictors considered in the 4 models.

**ANSWER: We did it. The same concern was raised by another reviewer. As suggested, we made explicit the variable to which lines b0, b1, b2, and b3 in Table 1 refer: the intercept, the literacy variable, the worldviews, and the interaction, respectively.**

4. In addition, I think it would be useful to do an analysis to indicate which of the 4 tested models is the best (e.g., ANOVA or AIC).

**ANSWER: We included AIC values in the last row of Table 1 and commented on them in the text as follows:**

***"*** ***The last row of Table 1 shows the AIC (Akaike information criterion) values for the four fitted models. The model with the lowest AIC included total literacy as the explanatory variable, while the model with the highest AIC included numeracy as the explanatory variable. The difference between these two AICs was 7.651, indicating, according to Burnham and Anderson*** (2004)***, good support for considering the model that included total literacy as an explanatory variable as a better model. A similar result, even if with weaker evidence, was found when considering the AIC difference between this model and those which included economic literacy (3.137) or science literacy (5.658) as the explanatory variable"***

5. Further, I think it would be interesting, also for the practical implications of the study, to have a model that simultaneously estimates the effect of numeracy, economic, and science literacy, unless they have a strong correlation with each other (in which case it should be specified).

**ANSWER: Yes, the request makes sense. On the one hand, the three variables are quite related:**

```
> x1 <- df$economic
> x2 <- df$numeracy
> x3 <- df$science
> tmp <- cbind(x1,x2,x3)
> round(cor(tmp),3)
      x1    x2    x3
x1 1.000 0.457 0.588
x2 0.457 1.000 0.447
x3 0.588 0.447 1.000
```

**On the other, we tried to fit them all into a model (including the 3 interactions with z -worldviews). The only significant interaction is with x2 (numeracy); the variable x3 (science) is not significant (when the other two are included), while x1 (economic) is significant as a main effect.**

**(note, only significant interactions are shown here, except for the variable included in the interaction)**

```

            Estimate Std. Error t value Pr(>|t|)
(Intercept)  2.94944    0.19247  15.324  < 2e-16 ***
z           -0.15534    0.20395  -0.762  0.44658
x2           0.09704    0.05095   1.904  0.05738 .
x1           0.03143    0.01210   2.597  0.00966 **
z:x2        -0.15095    0.05874  -2.570  0.01044 *

```

**Results show that:**

**When z = 0 we have that the expected values are given by 2.95 + 0.031xeconomic + 0.097xnumeracy**

**When z = 1 we have that the expected values are given by (2.95-0.16) + 0.031xeconomic + (0.097-0.15)x numeracy. Thus, with economic being equal, as numeracy increases, the predicted values in the group with z=0 grow, while in the group with z=1, again with economic being equal, as numeracy increases, the predicted values decrease (or rather, remain essentially constant).**

**In both groups (z=0 and z=1), with equal numeracy, as economic increases, the expected values increase (in the same way in both groups). In both groups (z=0 and z=1), at equal numeracy and economic, the predicted values do not depend on science.**

**Given these results, we believe it is more elegant to show a separate model for each predictor. Indeed, the purpose of our work was not to have a competition among predictors to see which among the three predictors was more powerful in determining the effect but rather to observe how each determined the effect. The three predictors, in our view, measure related but also very different, constructs, otherwise, we would not have studied all three of them. In the introductory part of the paper we expand on each of them by noting how each has its own distinctive features in relation to the immigration issue. In conclusion, we believe the proposed solution with the three predictors examined in three separate models is better. However, if the editor prefers, we can easily edit or include also this analysis in the manuscript.**

6. Further, it should be made explicit how the Total literacy item was calculated.

**ANSWER: Sorry if we weren't clear enough. The total literacy variable was calculated as the mean of the aggregated knowledge measures. We specified this in the methods section.**

7. - Page 21: Why the mean values of the variable perception of immigration was only estimated for numeracy? I think it should be interesting to consider also economic and science literacy.

**ANSWER: Reviewer 1 says that Table 2 basically replicates the data already presented in Figure 2 and says to remove it. You tell us to add the other data for science, and economic. We decided that it was better to remove it because it does not tell something different than Figure 2.**

8. - Since you decide to investigate cultural worldview instead of using political orientation, I would suggest discussing it more in deep. For example, are there any studies investigating the correlation between the two measures? This could help support your choice of not considering the political orientation and polarization, even if the Italian political situation is very "confused". I suggest that the link between polarization and cultural worldview is discussed more fully already in the introduction.

**ANSWER: Sorry for the confusion. We did write that we decided to investigate cultural worldview instead of using political orientation but we did not mean it, literally. To be more clear, we choose to measure worldviews not as a "substitute" or "proxy" of political worldviews but because we believed that they were better suited to measure knowledge-related polarization, than political orientation, when dealing with attitudes and behaviors in front of threats (such as immigration). As you suggested we now have detailed our arguments in the paper, both in the introduction and in the conclusion sections.**

**In the introduction:**

***Most studies on the polarization of beliefs used political orientation as the polarizing variable. Instead, following Kahan et al. (2012), we used a more nuanced underlying ideological measure, i.e., worldviews, to capture where the individual lies on the ideological spectrum represented by hierarchical-individualistic views on one side and egalitarian-communitarian views on the other. Individual preferences on social problems such as gun control, nuclear waste disposals, COVID-19, and climate change are strongly influenced by cultural worldviews*** (Cherry et al., 2017; Dryhurst et al., 2020; Kahan et al., 2011; Siegrist & Bearth, 2021)***. Indeed, in a study of 6,991 individuals across the world, an individualistic worldview predicted COVID-19-related attitudes and behaviors more than all other variables (including political orientation) in five out of the ten countries surveyed (UK, Germany, Sweden, Spain, and Japan)*** (Dryhurst et al., 2020)***. Moreover, worldviews have been successfully used in a prior study on risk perception and the polarizing impact of knowledge (Kahan, 2012). We, therefore, measured individual worldviews to categorize individuals into hierarchical-individualistic vs. egalitarian-communitarian and measure the polarizing impact that this underlying ideology induces when put in interaction with personal knowledge***.***.***

**In the conclusions:**

***"*** ***Many studies have used a measure of political orientation to elicit the knowledge-related polarization effect*** (Drummond & Fischhoff, 2017; Hamilton et al., 2015; Smith et al., 2017)***. While in some cases this has been a pre-designed choice*** (e.g., Shoots-Reinhard et al., 2021)***, in other cases such as large-scale representative surveys, it has been an ex-post forced choice due to its availability*** (e.g., Drummond & Fischhoff, 2017)***. In deciding the ideological measure to use in our study we decided to avoid using political orientation. Political orientation is typically elicited by asking respondents to classify themselves on some bipolar dimension, such as, republican vs. democrat or liberal vs. conservative. Instead, we preferred to use a measure of cultural worldviews*** (Kahan et al., 2011)***. The reason for this choice was twofold. On the one hand, a standard question about political orientation (right-wing or left-wing) would not adapt well to our context. Indeed, the Italian political landscape is characterized by small and fragmented parties with transversal positions with respect to the standard right-wing or left-wing dichotomy. For example, the 5 Star Movement is a populist party difficult to classify as right or left*** (Roccato et al., 2020; Verbeek & Zaslove, 2016)***. It has both right-wing (e.g., anti-immigrant) and left-wing (e.g., guaranteed minimum income) ideologies, as well as both conservative (e.g., NO TAV movement) and liberal (e.g., drug liberalization) ideologies*** (Roccato et al., 2020)***. A second and more important reason is that we believe worldviews are a more detailed and nuanced measure of the underlying ideology of individual beliefs and behaviors than political orientation, with whom they do not fully overlap. Worldviews capture where an individual stands on the spectrum anchored by hierarchical-individualistic beliefs at one pole and egalitarian-communitarian beliefs at the other. They have proved to be successful in identifying a knowledge-related polarizing effect in the case of previous risk perception studies (Kahan 2912). Moreover, worldview orientations have been shown to be significant predictors of an individual’s attitudes and behaviors in the face of threats*** (Chen et al., 2020; Siegrist & Bearth, 2021; Xue et al., 2014)***, sometimes even more than political orientations*** (Dryhurst et al., 2020)***. Our study confirms that cultural worldviews may be a valid construct for measuring knowledge-related polarization effects of risk perceptions of social problems***

9. - Page 19 line 11: I think is row b2 and not b3. In addition, compared to the results presented, I suggest a more cautious interpretation of the non-significant main effect of the z variable.

**ANSWER: Yes thank you, you are right, here was a mistake: b3 should have been b2; we have now corrected it.**

10. - I would not use x, y, and z in the main text, I think it makes the reading not very smooth.

**ANSWER: Thank you for the advice. Indeed, you are right. We have deleted references to x, y and z in all sections of the manuscript, as well in the figure and table captions, except for the section on "data analysis".**

11. - Figure 2 caption is quite long and quite overly descriptive.

**ANSWER: We reduced the length of the caption sensibly by eliminating the reference to x, z and y, shortening the text, but if we need to shorten it further, we can do it by moving part of the caption into the main text if necessary. Please just ask us.**

12. - In the state of the art, I would anticipate (briefly) why the numeracy, economic, and science literacy scales were chosen as variables. I believe that this clarification can be helpful in the reading.

**ANSWER: This was an excellent advise, thank you. In the State of the Art we have now included a paragraph which anticipates the decision to measure the three predictors (science, economic and number literacy). The paragraph is the following:**

***"*** ***Of central importance to the knowledge-related polarization effect is the type of knowledge/education/ability considered in the interaction*** (Shoots-Reinhard et al., 2021)***. Across studies on polarization, researchers have mostly used education as the knowledge variable to test for the interaction between partnership and opinions, with few exceptions that used science knowledge and others that used cognitive abilities (i.e., numeracy and verbal abilities). The type of knowledge variable chosen has been shown to determine the chance of detecting the interaction with ideology*** (Shoots-Reinhard et al., 2021)***. When verbal ability measures were not controlled for, numeracy and ideology did interact to predict outcomes, but they failed to interact when the verbal ability measure was included in the model*** (Shoots-Reinhard et al., 2021)***. Thus in the present study, we chose to examine the hypothesized interaction effect (knowledge x ideology), exploring multiple knowledge variables. We used two knowledge predictors (science and economic literacy) and one cognitive ability predictor (numeracy) to broaden the set of knowledge variables that elicit the knowledge-related polarization effect.***

***."***

13. - About science literacy (paragraph 2.4), I would add some references (also from newspapers) to justify the passage on the intertwining between migrants and the increase of viral infections.

**ANSWER: You're right, we make a lot of statements but don't support them with adequate references. We have now added references. We believe the paragraph is much improved now:**

***"*** ***Science literacy is the knowledge of basic scientific facts*** (National Science Board & National Science Foundation, 2020)***. While scientific knowledge is usually not a significant predictor of risk perception per se*** (Ho et al., 2019; Kahan et al., 2012)***, it is a significant factor in polarizing public opinions about climate change*** (Drummond & Fischhoff, 2017; Kahan et al., 2012)***. Individuals with higher science literacy showed the greatest cultural-worldviews polarization for climate change risks*** (Kahan et al., 2012)***. Seemingly, individuals with greater science knowledge showed more political polarization on issues such as stem cell research, the big bang, human evolution, and climate change*** (Drummond & Fischhoff, 2017)***. On a similar line, greater attention to scientific news increased support for policies aimed at reducing climate change for strong liberals but reduced support for strong conservatives*** (Hart et al., 2015)***. Science knowledge and fear of immigration share a common ground when it comes to viruses and diseases. Indeed, people might fear immigrants thinking that they can be vehicles for viruses and diseases. Concerns about immigrants and disease have been constantly registered throughout history. For example, Merkel and Stern*** (2002) ***explored why in three periods, from 1880 to the present, immigrants have been stigmatized as the etiology of a variety of diseases, despite the data do not support such a narrative. Human mobility, indeed, was historically associated with the spread of infectious diseases*** (Castelli & Sulis, 2017)***, however this relationship no longer existing in the contemporary age. Despite this, fear is still supported by the media who portray immigrants as disease spreaders*** (Esses et al., 2013)***. For example, during the of COVID-19 outbreak in Italy, the question of whether more immigrants should be brought into the country from the sea borders was also deeply intertwined with the threat they posed as positive drivers of viral infection*** (Rowe et al., 2021)***. However, this might be especially true for individuals opposing immigration who historically hold a more ideological attitude of right-wing authoritarianism. We, therefore, predicted that greater science knowledge would interact with cultural worldview orientations in explaining public opinion on immigration. Thus, we anticipated those people with greater science knowledge, and egalitarian-communitarian orientation might show more extreme positive opinions on immigration, while those people with greater science knowledge and hierarchical-individualistic orientation might show more strong negative opinions on immigration. “***

14. - I suggest reviewing the Supplementary Materials, in some of the presented scales references are specified in others not. Additionally, in the Perception of immigration, I believe it would be appropriate to differentiate items from the 1974/2014 General Social Survey (Smith et al., 2017) from those adapted from other work.

**ANSWER: The supplementary materials have been enriched with new tables and analyses. We have standardized the references and we have also modified, as you suggested, the table of items for measuring perceptions of immigration by inserting an asterisk to indicate those items that were taken from the GSS. Thanks for the suggestions and thanks for your careful reading.**

15. - Page 23: There was some problem with the citation of Roccato et al., 2020

**ANSWER: Yes, thank you we fixed it. Mendley did something weird with this entry.**

16. - As for the language, I am not a native English speaker, so I will defer to others in evaluating this aspect.

**ANSWER: We, too, are not native English speakers. We used proofreading software to double-check the wording, but if required, we may have the paper reviewed by a private proofreading agency.**
